# Treatments for Alzheimer’s and the declaration of Helsinki

**DOI:** 10.1016/j.tjpad.2025.100260

**Published:** 2025-06-27

**Authors:** Timothy Daly, Andi Olluri, Markku Kurkinen

**Affiliations:** a UMR 1219 Bordeaux Population Health, Université de Bordeaux & INSERM, 146 rue Léo Saignat, 33076 Bordeaux, France; b UMR 5164, ImmunoConcept, University of Bordeaux & CNRS, 2 Rue Dr Hoffmann Martinot, 33076 Bordeaux, France; c Bioethics Program, FLACSO Argentina, Tucumán 1966, C1050 AAN, Buenos Aires, Argentina; d Student at Sahlgrenska Academy, University of Gothenburg, Sweden; e NeuroActiva, Inc., San Jose, USA

In *The Journal of Prevention of Alzheimer’s Disease,* Hsu et al. [1] close their systematic review and meta-analysis on treatments for Alzheimer’s disease (AD) with three major conclusions. First, compared to placebo, only anti-amyloid monoclonal antibodies (mABs) and not acetylcholinesterase inhibitors (AChEIs) were associated with a slowing of cognitive decline. Second, though not based on “head-to-head” comparisons within a randomized clinical trial (RCT), mABs outperformed AChEIs. Third, the slowing effects of mABs do not reach clinical significance. Their study adds to a growing literature on the analysis of mABs and comparisons with AChEIs along the lines of efficacy, safety, and medico-economic assessment (see references 47—55 of Hsu et al. [1]). We will discuss each of their conclusions in the light of data analysis and the Declaration of Helsinki (DoH), a set of principles concerning health-related research with humans.

All mAB trials with anti-amyloid antibodies claimed to follow the DoH [2] in their Methods section, but here we will focus on the donanemab trial, whose full protocol for “Study I5T-MC-AACI (AACI)” with donanemab is publicly available online as Supplementary Material [3]. The DoH, which was first published by the World Medical Association (WMA) in 1964 and named after the city where it was first finalized, extended the 1947 Nuremberg Code and the 1948 Declaration of Geneva to focus on the ethics of clinical research. It remains the foundational living framework for protections in health-related research involving human participants and its updated 2024 version comprises 37 articles developed through a process of proposed changes, broad stakeholder consultations, iterative draft revisions, international debates, text negotiations, and formal WMA approval. Protections include respect for persons, a meaningful informed consent process, rigorous risk-benefit assessments, safeguards for participants in situations of vulnerability, and independent ethical review of all research protocols. These principles also extend beyond clinical trials to inform ethical medical practice globally. We analyze mAB trials in AD in light of the use of placebo mentioned in the DoH 2013 [2], the version available at the time of the donanemab trial.

## 1 Hsu et al. conclusion one: only mABs, not AChEIs, outperform placebo

Both 18 month RCTs of lecanemab and donanemab compared to placebo used the Clinical Dementia Rating Sum of Boxes (CDR-SB) scale, a measure of cognition and behavior ranging from 0 to 18. Lecanemab slowed cognitive decline by 0.45 points on, frequently and incorrectly interpreted in both scientific and lay media as representing a 27 % slower decline; however, when accounting for baseline scores, the true absolute difference is closer to 9.3 %. Importantly, this 0.45-point change falls below the CDR-SB’s minimum observable unit of 0.5, rendering the reported effect statistically detectable but clinically meaningless. Both lecanemab and donanemab trials used weight-adjusted averages, which obscure variation between subgroups (e.g., sex or genotype), producing summary figures that do not represent any real patient population. Lecanemab showed notably lower efficacy for women and APOE4 carriers, who make up the vast majority of the AD population [4]. These subgroup results were buried in supplementary appendices. In the donanemab trial, a 0.68-point difference in CDR-SB score change between the donanemab and placebo groups was widely interpreted as a 36 % slowing of cognitive decline. However, the correct absolute difference is 9.6 % less cognitive impairment, comparable to lecanemab. Similarly, when cognition and behavior were measured using the Integrated Alzheimer’s Disease Rating Scale (iADRS), a 144-point scale where lower scores indicate worse impairment, the 3.25-point difference between groups was reported as a 35 % slowing of decline. This corresponds to a 2.5 % absolute reduction in impairment with donanemab compared to placebo.

## 2 Hsu et al. conclusion two: mABs outperform AChEIs

Hsu et al. claim that mABs outperform AChEIs [1]. But this second conclusion is not based on “head-to-head” comparisons of randomized clinical trial (RCTs) between mABs and AChEIs [4]. This absence of direct comparison may not seem problematic for those who follow the dominant reading that emphasizes the fact that AChEIs are symptomatic treatments, whereas mABs are disease-modifying treatments (DMTs), so why compare them? This point is mentioned in the DoH 2013. We will see that there are two criteria justifying the non-use of placebo in RCTs of a new treatment. In this case, donanemab satisfies the “no-harm” criterion, but not the “interference” criterion. According to paragraph 33 of DoH 2013 [2]:

“*33. use of placebo*

*The benefits, risks, burdens and effectiveness of a new intervention must be tested against those of the best proven intervention(s), except in the following circumstances:*

- *Where no proven intervention exists, the use of placebo, or no intervention, is acceptable; or*
- *Where for compelling and scientifically sound methodological reasons the use of any intervention less effective than the best proven one, the use of placebo, or no intervention is necessary to determine the efficacy or safety of an intervention and the patients who receive any intervention less effective than the best proven one, placebo, or no intervention will not be subject to additional risks of serious or irreversible harm as a result of not receiving the best proven intervention.*

*Extreme care must be taken to avoid abuse of this option.*”

There is an ethical ambiguity when there seem to be two different classes of drugs: approved AChEIs (thought to be symptomatic), versus mABs (thought to be DMTs). In this case, the dilemma is as follows: to respect para. 33, can trial sponsors of a potential DMT for AD use a placebo control, or must they test the potential DMT head-to-head against AChEIs?

According to our reading of para. 33, DoH 2013 allows for not using the best standard-of-care symptomatic treatment (AChEIs) only under two conditions: scientific necessity (i.e. the symptomatic treatment might interfere with measuring disease progression), and minimal risk of serious or irreversible harm to participants (i.e. withholding symptomatic relief will not cause significant suffering or deterioration). For clarity, we will call these two criteria the “interference” criteria and the “no-harm”.

Let us now turn to the justification for use of placebo in the donanemab trial [3] (Supplementary Trial Protocol, p.41):

*Study AACI includes a placebo treatment arm and allows all participants to continue their symptomatic AD standard of care concomitant medications. Inclusion of a placebo treatment arm is acceptable because there are no confirmed clinically effective disease modifying treatments for AD; this approach is in agreement with the use of placebo described in the Declaration of Helsinki (WMA 2013). The use of a placebo comparator in Study AACI is needed to determine the efficacy and safety of donanemab therapy*.

Does this justification meet the “interference” and “no-harm” criteria? AChEI use was part of the randomization criteria of the study, and did not lead to a significant reduction in the use of AChEI between the control and placebo groups. Thus, the above justification satisfies the “no-harm” criteria. Does it satisfy the “interference” criterion? The protocol identifies that there is no proven DMT for AD, but neglects to justify that “to determine the efficacy and safety”, placebo is scientifically necessary. There is no discussion of why an active comparator trial (e.g., donanemab vs standard care alone) would not suffice, or how placebo is essential to separate true drug effect from background noise. Thus, we consider that the donanemab trial sponsors have not justified placebo use with the “extreme care” stipulated in DoH 2013.

## 3 Hsu et al. conclusion three: mABs do not reach clinical meaningfulness

Hsu et al. correctly claim that the effects of mABs on slowing cognitive decline associated with AD does not reach the threshold of clinical meaningfulness. Given mABs’ cost, their lack of impact on clinically-meaningful outcomes in AD, and their side-effect profile, their approval might not represent a gain for individuals or health systems [5]. Nevertheless, many of the claims made about the effects of mABs in AD are framed using the language of meaningful measures for individuals (e.g. having more time to spend with grandkids), despite the fact that clinical trial data do not support this claim [4]. This raises ethical issues related to right communication in science and medicine.

## 4 Conclusion

mABs are thought to be DMTs for AD, but they were not tested head-to-head against approved AChEIs in RCTs, but rather against placebo. We consider that this choice is not fully justified in light of DoH 2013, para. 33: while it may not have caused harm to patients, the scientific justification for placebo was incomplete (drug sponsors did not adequately demonstrate or even argue that AChEIs would interfere with measurements of disease modification). Furthermore, Hsu et al.’s Conclusion One provides a scientific argument for the non-interference of AChEIs in trials of mABs. Therefore, we consider that any further testing of the value of mABs to patients with AD should involve head-to-head RCTs between mABs and AChEIs [4].

## CRediT authorship contribution statement

**Timothy Daly:** Conceptualization, Validation, Visualization, Writing – original draft, Writing – review & editing. **Andi Olluri:** Validation, Visualization. **Markku Kurkinen:** Validation, Visualization, Writing – review & editing.

## Declaration of competing interest

The authors declare that they have no known competing financial interests or personal relationships that could have appeared to influence the work reported in this paper.
